# Prognostic Significance of the Royal Marsden Hospital (RMH) Score in Patients with Cancer: A Systematic Review and Meta-Analysis

**DOI:** 10.3390/cancers16101835

**Published:** 2024-05-11

**Authors:** Taha Koray Sahin, Alessandro Rizzo, Sercan Aksoy, Deniz Can Guven

**Affiliations:** 1 Department of Medical Oncology, Hacettepe University, Ankara 06100, Turkey; takorsah@gmail.com (T.K.S.); saksoy07@yahoo.com (S.A.); 2 IRCCS Istituto Tumori “Giovanni Paolo II”, 70124 Bari, Italy; rizzo.alessandro179@gmail.com; 3 Medical Oncology Clinic, Health Sciences University, Elazig City Hospital, Elazig 23280, Turkey

**Keywords:** prognostic score, Royal Marsden Hospital score, survival, meta-analysis, cancer

## Abstract

**Simple Summary:**

Despite the promising evidence of the Royal Marsden Hospital (RMH) score as a readily available prognostic biomarker in patients with cancer, the wide scale implementation in clinical practice as well as the true benefit in clinical decision-making is lacking. Therefore, we systematically reviewed the available evidence on the association between the RMH score and prognosis in patients with cancer. This comprehensive meta-analysis, encompassing over a hundred thousand patients, revealed a negative association between a higher RMH score and survival in cancer patients. The available evidence demonstrates that the RMH score is not only a selective biomarker for patients enrolled in clinical trials, but also a useful prognostic biomarker in a real-world setting. Future research should aim to validate and refine this score, ensuring its optimal application in clinical practice and decision-making.

**Abstract:**

Background: Cancer remains a leading cause of death globally, necessitating the identification of prognostic biomarkers to guide treatment decisions. The Royal Marsden Hospital (RMH) score, based on readily available blood tests and clinical features, has emerged as a prognostic tool, although its performance across variable clinical scenarios is not thoroughly delineated. Therefore, we aimed to systematically assess the association between RMH score and survival in cancer patients. Methods: We conducted a systematic literature search across Pubmed, Scopus, and Web of Science databases for studies published up to 15 February 2024. We performed a meta-analysis with the generic inverse variance method with a random-effects model and reported hazard ratios (HR) with 95% confidence intervals (CI). Results: Nineteen studies encompassing 127,230 patients were included. A higher RMH score was significantly associated with worse overall survival (OS) (HR: 2.09, 95% CI: 1.87–2.33, *p* < 0.001) and progression-free survival (PFS) (HR: 1.80, 95% CI: 1.48–2.18, *p* < 0.001). This association was consistent across various subgroups, including study population (clinical trial vs. real-world cohort), geographic region, and tumor type. Conclusion: This meta-analysis, including over a hundred thousand patients, demonstrates a negative association between a higher RMH score and survival in cancer patients. The RMH score holds promise as a readily available prognostic tool across diverse cancer types and clinical settings. Future research should focus on validating and refining this score to aid clinical decision-making.

## 1. Introduction

Cancer is the second most common cause of mortality worldwide, with almost 20 million new patients and over 10 million deaths per year [1]. Despite advances in the diagnostic and therapeutic fields, over 50% of patients with cancer eventually die of the disease [2]. Additionally, the survivors deal with a wide array of morbidities caused by the disease itself, as well as treatment-related toxicities [3]. These points altogether denote a need for biomarkers aiding in treatment escalations in patients with a poorer prognosis and de-escalations to mitigate toxicities in patients with a lower-risk disease. However, biomarker research has lagged, possibly due to lower pharma interest, problems with tissue availability, and the need for complex platforms for most prognostic biomarkers.

A growing body of research underscores the crucial role of patients’ inflammatory and nutritional status on cancer progression, treatment response, and cancer survival [4]. The significance of inflammation and nutritional status in cancer prognosis has led to the identification and utilization of several biomarkers, including the prognostic nutrition index (PNI), neutrophil-to-lymphocyte ratio (NLR), and pan-immune-inflammation value (PIV) [5,6]. Similar to these existing inflammation/nutrition-based biomarkers, one such biomarker gaining attention, the Royal Marsden Hospital (RMH) score, utilizes readily available blood tests [6]. The RMH prognostic score, which includes albumin level, lactate dehydrogenase (LDH) level, and number of metastases, was initially introduced by Arkenau et al. as a prognostic score to optimize patient selection for phase I clinical trials, often involving highly selected patient groups [7]. They found that patients with a low RMH score (0–1) had a significantly longer median overall survival (OS) of 33 weeks compared to 15.7 weeks for patients with a high RMH score (2–3). After that, the prognostic value of the RMH score for predicting outcomes was explored for various cancers on phase 1 trial cohorts, as well as a few cohorts from real-world settings [8,9]. However, this raises questions about the RMH score’s generalizability in clinical trials to the larger and more variable patient populations encountered in daily clinical practice, necessitating a review of the available evidence.

Despite the promising evidence of the RMH score as a readily available prognostic biomarker in patients with cancer, the available studies differed in study design, study population (clinical trial vs. real-life cohorts), and sample sizes, limiting the individual study’s generalizability. Therefore, we systematically reviewed the available evidence on the association between the RMH score and prognosis in patients with cancer.

## 2. Materials and Methods

### 2.1. Literature Search

We conducted a systematic review following the Preferred Reporting Items for Systematic reviews and Meta-Analyses (PRISMA) guidelines [10]. This protocol has been registered on the PROSPERO database (Registration Number: CRD42024530801). Then, a comprehensive search strategy was performed across the Web of Science, PubMed, and Scopus, encompassing the relevant literature published up to 15 February 2024. The following search terms were used: “Royal Marsden Hospital” AND “score” AND “cancer”. Furthermore, a manual search of reference lists from included articles was performed to identify any additional relevant studies. To minimize bias and enhance the reliability of the search process, two independent investigators (T.K.S. and D.C.G.) performed the literature search.

### 2.2. Inclusion and Exclusion Criteria

We included studies that met the following inclusion criteria: (1) retrospective or prospective studies investigating the association between RMH score and progression-free survival (PFS) or overall survival (OS); (2) definition of RMH score risk groups as in the pivotal paper (0–1 point: low risk, 2–3 points: high risk); (3) available hazard ratios (HR) and 95% confidence interval for the comparison of low-RMH and high-RMH score groups; (4) full-text article or abstract available in English. Exclusion criteria of studies were: (1) duplicated publications; (2) case series, case reports, guidelines, editorials, or reviews; (3) categorization of RMH score risk groups differently than the standard criteria; (4) lack of HR and CIs for PFS or OS.

### 2.3. Data Extraction and Study Selection

**Figure 1:**
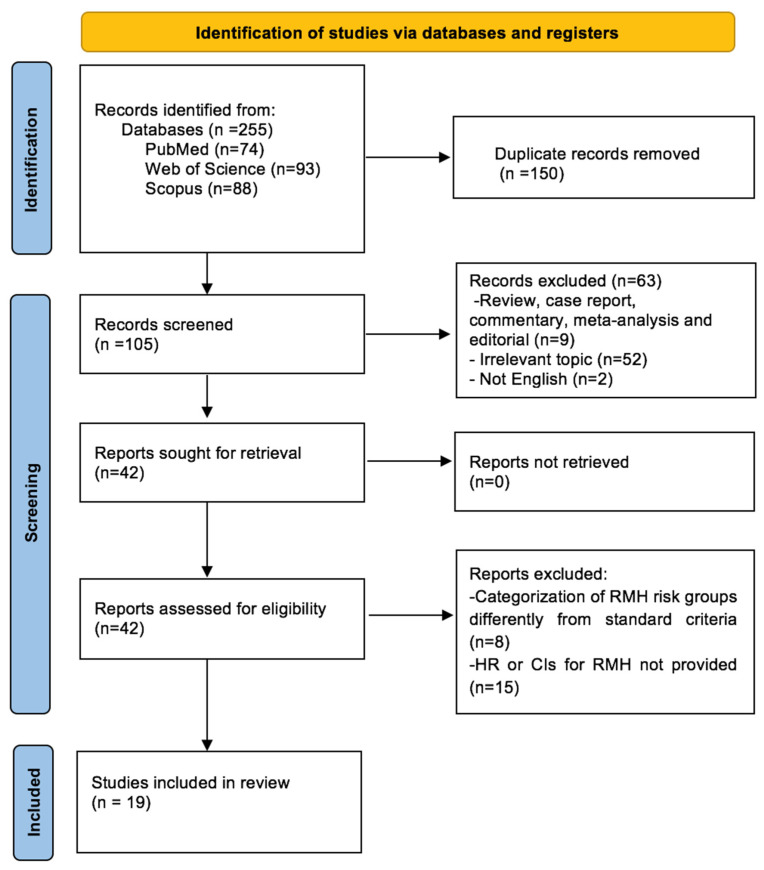
PRISMA flow diagram for article selection.

This initial systematic search yielded a total of 255 records. After the removal of duplications (n = 150), we screened the remaining 105 records based on their titles and abstracts. A total of 63 records were excluded due to irrelevant topic (n = 52), article type (meta-analyses, review, editorial, or case report) (n = 9), and non-English language (n = 2). Eight more records were excluded due to categorization of RMH score risk groups differently from standard criteria and no available HR and CIs for survival (n = 15); thus, a total of 19 studies was deemed eligible for inclusion in the systematic review. A detailed flowchart depicting the article selection process is presented in Figure 1.

Two independent authors (T.K.S., D.C.G.) performed data extraction adhering to the Meta-analyses of Observational Studies in Epidemiology (MOOSE) guidelines [11]. The following information was extracted from the available studies: name of lead author, publication year, the total number of patients, number of patients in low or high-risk RMH score groups, country, tumor type, type of treatment, disease stage, HR with 95% CIs for PFS or OS. Whenever available, HR data derived from multivariable analyses were prioritized for extraction. Methodological quality and risk of bias were assessed independently by two authors (T.K.S. and D.C.G.) using the Newcastle–Ottawa Scale.

### 2.4. Meta-Analysis

The primary objective was to evaluate the association between RMH score and OS in patients with cancer. The secondary objective was to evaluate the association between the PFS and the RMH score. Subgroup analyses were conducted according to country (USA and Canada, Europe, Asia), study cohort characteristics (clinical trial vs. real-world cohort), and tumor type.

Meta-analyses were carried out using the generic inverse variance method with the random-effects analysis model to minimize potential heterogeneity in the analyses. HRs and 95% CIs were employed as the principal summary measure. The assessment of heterogeneity was conducted within each subgroup using I2 statistics, with I2 values over 25% indicating significant heterogeneity. Review Manager software (version 5.4; Nordic Cochrane Center, Copenhagen, Denmark) was employed to conduct the meta-analyses. A *p*-value of 0.05 was adopted as the level of statistical significance.

## 3. Results

### 3.1. Study Characteristics

The main summary characteristics of the included studies are shown in Table 1. This meta-analysis included a total of 19 studies, encompassing 127,230 patients. The available records were published from 2009 to 2023. Most studies (n = 17) had retrospective designs and primarily enrolled patients from phase I clinical trials (n = 14). Fifteen studies were single-center, whereas four had multicenter designs. Geographically, nine out of nineteen studies presented data from North America, followed by six and four papers conducted in Europe and Asia, respectively. The available studies were conducted in several tumors, including colorectal cancer, sarcoma, small cell lung cancer, and non-small cell lung cancer. Additionally, over half of the studies included a basket cohort consisting of several types of tumors (n = 10). Eighteen studies reported OS data, while six studies provided PFS data. The Newcastle–Ottawa Scale (NOS) was used to assess methodological quality, with most studies demonstrating a low risk of bias (Table 2).

### 3.2. Association between RMH Score and OS

**Figure 2:**
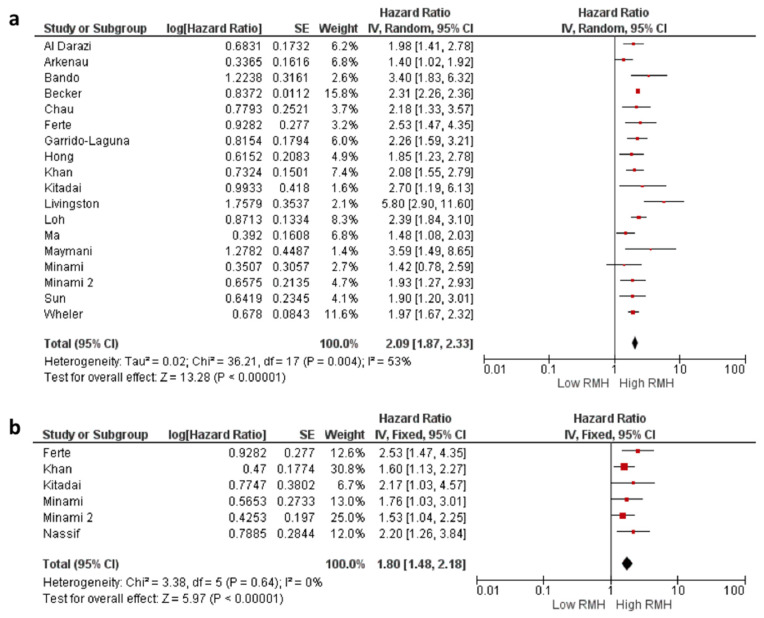
The association between the Royal Marsden Hospital (RMH) score and overall survival (**a**) and progression-free survival (**b**). CI = confidence interval; I^2^ = heterogeneity; df = degrees of freedom; SE: standard error. Each red square in Figure 2 represents an effect size of a study and the area of the square represents the magnitude of a related study in the effect size. The lines on either side of the squares indicate the lower and upper limits in a 95% CI of the calculated effect sizes. The black rhombus at the bottom of the plot shows the calculated overall effect size.

All but one study reported a negative effect of high RMH score on OS. In the pooled analysis of eighteen studies, the risk of death was higher in patients with a high RMH score compared to a low RMH score (HR: 2.09, 95% CI: 1.87–2.33, *p* < 0.001) (Figure 2). The included studies had a moderate degree of heterogeneity (I2 = 53%). Sensitivity analyses conducted by subtraction of the individual studies demonstrated consistent results.

**Figure 3:**
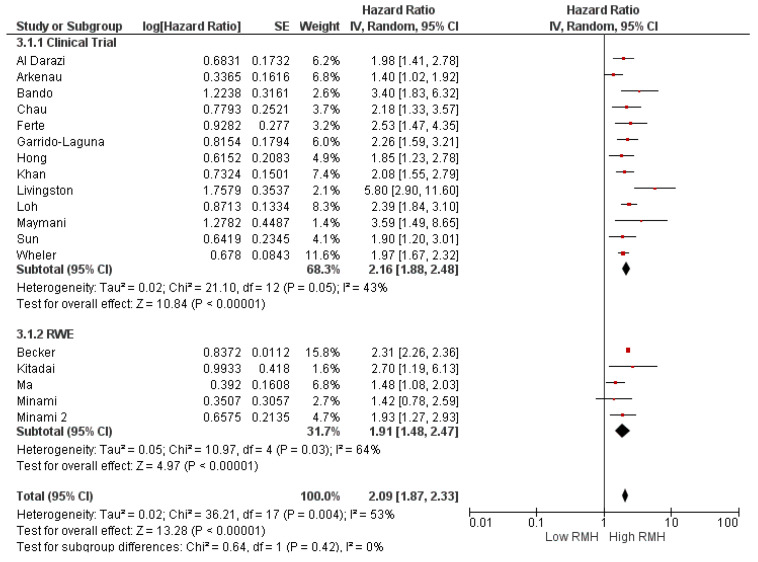
Subgroup analyses of overall survival according to study population (clinical trial and real-word evidence (RWE)). CI = confidence interval; I^2^ = heterogeneity; df = degrees of freedom; SE: standard error. Each red square in Figure 2 represents an effect size of a study and the area of the square represents the magnitude of a related study in the effect size. The lines on either side of the squares indicate the lower and upper limits in a 95% CI of the calculated effect sizes. The black rhombus at the bottom of the plot shows the calculated overall effect size.

**Figure 4:**
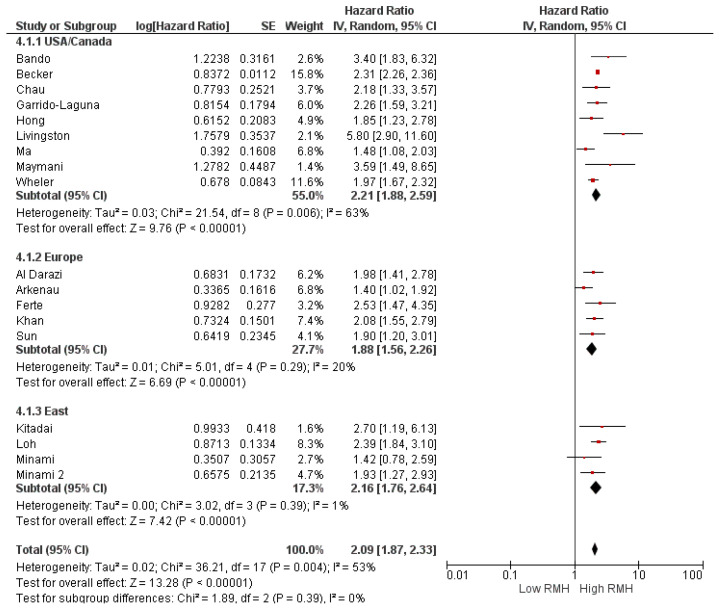
Subgroup analyses of overall survival according to country. CI = confidence interval; I^2^ = heterogeneity; df = degrees of freedom; SE: standard error. Each red square in Figure 2 represents an effect size of a study and the area of the square represents the magnitude of a related study in the effect size. The lines on either side of the squares indicate the lower and upper limits in a 95% CI of the calculated effect sizes. The black rhombus at the bottom of the plot shows the calculated overall effect size.

**Figure 5:**
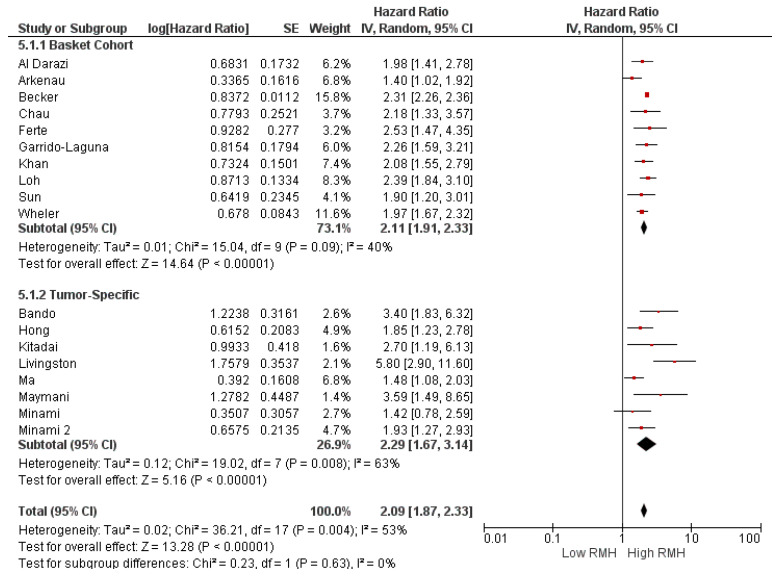
Subgroup analyses of overall survival according to cancer type (basket cohort and tumor-specific). CI = confidence interval; I^2^ = heterogeneity; df = degrees of freedom; SE: standard error. Each red square in Figure 2 represents an effect size of a study and the area of the square represents the magnitude of a related study in the effect size. The lines on either side of the squares indicate the lower and upper limits in a 95% CI of the calculated effect sizes. The black rhombus at the bottom of the plot shows the calculated overall effect size.

Subgroup analyses for the study population (clinical trial (HR: 2.16, 95% CI: 1.88–2.48, *p* < 0.001) and real-world evidence (HR: 1.91, 95% CI: 1.48–2.47, *p* < 0.001) demonstrated a similar association between high RMH score and OS (Figure 3). Similarly, subgroup analyses according to study location (HR: 2.21, 95% CI: 1.88–2.59, *p* < 0.0001 for North America, HR: 1.88, 95% CI: 1.56–2.26, *p* < 0.0001 for Europe, and HR: 2.16, 95% CI: 1.76–2.64 *p* < 0.0001 for Asia) (Figure 4) and cancer type (HR: 2.11, 95% CI: 1.91–2.33, *p* < 0.001 for basket cohort, and HR: 2.29, 95% CI: 1.67–3.14, *p* < 0.001 for tumor-specific) demonstrated a consistently higher risk of death in patients with a high RMH score than in patients with a low RMH score (Figure 5).

### 3.3. Association between RMH Score and PFS

Data on PFS were available from six studies and included in the meta-analysis. In the pooled analysis, patients with a high RMH score had an increased risk of progression or death compared to those with a low RMH score (HR: 1.80, 95% CI: 1.48–2.18, *p* < 0.001). Additionally, the I^2^ statistic (I^2^ = 0%) indicated a lack of significant heterogeneity between the included studies, suggesting consistency across studies (Figure 2). 

## 4. Discussion

In this meta-analysis, we observed a negative association between a higher RMH score and OS or PFS. The adverse effect of a high RMH score in OS was consistent across geographic regions (USA and Canada, Europe, Asia), study cohort characteristics (clinical trial vs. real-world cohort), and tumor type. To the best of our knowledge, the present study is the first meta-analysis investigating the association between RMH score and survival outcomes in patients with cancer.

Inflammation is a fundamental hallmark of cancer that greatly contributes to oncogenesis and tumor progression [28]. Rampant inflammation and malnutrition play pivotal roles in cancer patients, resulting in poor treatment outcomes [29]. Mounting evidence demonstrates the utility of peripheral blood-based parameters as surrogate markers reflecting the inflammatory state and nutritional condition of cancer patients [30,31]. The minimally invasive retrieval, low cost, and quantitative nature of complete blood count (CBC)-derived biomarkers render them particularly advantageous for clinical application. Consequently, there is an intensifying focus on the use of biomarkers with demonstrable clinical relevance, aiming to integrate these metrics into the prognostic and therapeutic stratification of cancer patients.

The RMH score [32] is based on three objective parameters: albumin level (≥3.5 g/dL: 0 vs. <3.5 g/dL: 1), LDH level (normal: 0 vs. >upper limit normal: 1), and number of metastatic sites (≤2: 0 vs. >2: 1). Based on their total score, patients are categorized as high-risk (RMH score 2 or 3) or low-risk (RMH score 0 or 1) [7,12]. The RMH score was initially developed for optimal patient selection in early-phase clinical trials of new cytotoxic or biologic agents. Later studies were also mostly focused on these cohorts, further supporting the use of RMH in early phase clinical trials. In the prospective validation study of RMH score, leukocyte count was also identified as having a prognostic significance for OS; however, it was not included in the final scoring model [7]. Similar to previous studies that have demonstrated the utility of the RMH score in various types of cancer, including colorectal cancer, sarcoma, and non-small cell lung cancer (NSCLC) [14,18,21], Al Darazi et al. confirm the prognostic value of the score when applied to a population of varied tumor types, showcasing its widescale applicability in clinical trials encompassing various cancers [22]. The RMH score’s validity was further established in Asian populations participating in phase I clinical trials by Loh et al. [26]. Our analysis revealed a consistent association between a high RMH score and poorer overall survival across geographic regions, including North America, Europe, and Asia, further supporting the use of RMH scores in clinical practice.

Beyond clinical trials, Minami et al. demonstrated the baseline RMH score is an independent predictor of both OS and PFS in patients with extensive-stage small cell lung cancer (ES-SCLC), with the RMH score being the only independent prognostic factor for PFS [9]. Interestingly, the same study group found that the RMH score was an independent predictive marker for PFS, but not for OS, in patients with non-small cell lung cancer (NSCLC) receiving immunotherapy (ICI) therapy [8]. This could be due to the limited patient sample size and retrospective design of their study. Moreover, Becker et al. evaluated the prognostic role of RMH scores in a very large cohort encompassing over a hundred thousand patients in a real-world setting and reported shorter OS in patients with high RMH scores [23]. These findings highlight the RMH score’s potential as a prognostic tool, not only for selecting patients in clinical trials but also for patients in real-world clinical settings.

While direct comparisons between the RMH score and similar prognostic scores are scarce, Minami et al. investigated the Gustave Roussy Immune (GRIm)-Score alongside the RMH score in ES-SCLC [9]. Notably, the GRIm-Score incorporates the NLR instead of the number of metastases used in the RMH score, while retaining albumin and LDH as shared components [33]. Their study revealed that both the GRIm-Score and RMH score were independent prognostic factors for OS in patients with ES-SCLC. Alvarez et al. compared four prognostic scores (RMH, GRIM, MDAS, LIPI) to select patients for immunotherapy phase I trials. All four scores significantly predicted OS, but none showed statistically better ability compared to the others [34]. A recent study comprehensively compared the performance of six prognostic scoring systems for predicting survival in patients with cancer receiving ICI treatment in phase 1 trials [22]. Although the LIPI, MDACC, and GRIm scores showed better performance than the other scoring systems in predicting OS, further validation with larger cohorts and in real-world settings is necessary.

While the RMH score has been successful in predicting patient outcomes, there is ongoing research to improve its accuracy by incorporating additional factors. The study by Rodrigues et al. demonstrated that the addition of low skeletal muscle index and sex to the RMH score enhanced its ability to predict patient outcomes [35]. Sen et al. developed a novel prognostic score termed MDA-ICI, incorporating seven parameters for patients treated in phase 1 ICI clinical trials. This novel score demonstrated superior performance in predicting OS compared to the established RMH and MDACC scores [36]. The Harrell c-index of the MDA-ICI score was 0.72, while the C-indexes of the RMH and MDACC systems were 0.67 and 0.61, respectively. More recently, Loh et al. also developed a more comprehensive prognostic score, called the NCIS score [26]. This score combines the RMH score with two additional factors: Eastern Cooperative Oncology Group Performance Status (ECOG-PS) and tumor type. The NCIS score provides an excellent prediction of both short-term and long-term survival for patients in contemporary Phase I trials [26]. Additionally, the predictive power of the RMH score could be improved by incorporating the on-treatment changes. Our group recently developed a compound prognostic score (NLR2-CEL score) and demonstrated better AUC values for the prediction of OS compared to RMH score using both baseline and on-treatment NLR levels [37]. There should be an ongoing effort involving modifications to the original RMH score to include more clinical parameters as well as longitudinal studies exploring RMH score dynamics over time to improve prognostic accuracy of the RMH score. Encouraging international collaborations could facilitate large-scale studies and the development of a more robust prognostic tool across different clinical scenarios. Finally, comparing the RMH score with other available prognostic scores in future studies could establish its relative performance and potential advantages. Implementation of these steps could help in establishing the RMH score as a reliable, universally applicable prognostic tool in routine practice.

This meta-analysis has potential limitations. First, we relied on HRs and percentages reported from the studies rather than individual patient data. The included studies were heterogeneous regarding treatment, sample size, tumor type, and geographic region, although the subgroup analyses revealed a consistent trend across these clinically relevant subgroups. The generalizability of the RMH score is another concern, as most studies on the RMH score have been conducted within the confines of phase I clinical trials, which involve highly selected patient groups. Additionally, two of the parameters of the RMH score (LDH levels and number of metastatic sites) are generally correlated with the tumor burden. This issue could limit the RMH score’s potential to reflect immune-inflammatory status in addition to the tumor burden and malnutrition. Furthermore, two laboratory parameters included in the RMH score (albumin and LDH levels) are dichotomously categorized instead of continuous variable, possibly another contributor limiting the precision power of the RMH score. Lastly, most of these included studies were retrospective in design, which can introduce the risk of selection bias. However, despite these limitations, this meta-analysis offers the first comprehensive evaluation of a promising minimally invasive prognostic biomarker, the RMH score, in patients with cancer.

## 5. Conclusions

In conclusion, the available evidence demonstrates that the RMH score is not only a selective biomarker for patients enrolled in clinical trials, but also a useful prognostic biomarker in a real-world setting. Future research should aim to validate and refine this score, ensuring its optimal application in clinical practice and decision-making.

## Figures and Tables

**Table 1:** Baseline characteristics of included studies.

Study, Year	Country	Study Population	Cancer Type	Study Design	Sample Size (RMH Score Low–High) (n)	Primary Treatment	TNM Stage	Multivariate Analysis	Survival Outcomes	Median Follow Up
Arkenau, 2009 [7]	UK	Phase 1 Trials	Mixed	P;S	78 (43–35)	Mixed	Locoregional (%10) Metastatic (%90)	Multivariate	OS	27.3 weeks
Chau, 2011 [12]	Canada	Phase 1 Trials	Mixed	R;S	233 (191–42)	Mixed	Metastatic	Univariate	OS	224 days
Garrido-Laguna, 2011 [13]	USA	Phase 1 Trials	Mixed	R;S	229 (181–48)	Mixed	Metastatic	Multivariate	OS	N/A
Hong, 2012 [14]	USA	Phase 1 Trials	CRC	R;S	144 (82–62)	Mixed	Metastatic	Multivariate	OS	N/A
Wheler, 2012 [15]	USA	Phase 1 Trials	Mixed	R;S	1169 (908–261)	Mixed	Mixed	Multivariate	OS	8.13 months
Ferte, 2014 [16]	France	Phase 1 Trials	Mixed	P;M	188 (123–65)	Mixed	Metastatic	Multivariate	OS, PFS	N/A
Khan, 2016 [17]	UK	Phase 1 Trials	Mixed	R;S	315 (N/A)	Mixed	Metastatic	Multivariate	OS, PFS	N/A
Livingston, 2016 [18]	USA	Phase 1 Trials	Sarcomas	R;S	91 (74–17)	Mixed	Metastatic	Univariate	OS	N/A
Bando, 2017 [19]	USA	Phase 1 Trials	Esophagogastric	R;M	115 (86–29)	Mixed	Metastatic	Multivariate	OS	N/A
Sun, 2017 [20]	France	Phase 1 Trials	Mixed	R;S	167 (102–65)	ICI	Metastatic	Multivariate	OS	12.9 months
Maymani, 2018 [21]	USA	Phase 1 Trials	NSCLC	R;S	73 (67–6)	ICI	Metastatic	Univariate	OS	12.3 months
Minami, 2019 [8]	Japan	Real-World Cohort	NSCLC	R;S	74 (44–32)	ICI	Metastatic	Multivariate	OS, PFS	N/A
Minami, 2020 [9]	Japan	Real-World Cohort	SCLC	R;S	128 (54–74)	ICI	Metastatic	Multivariate	OS, PFS	N/A
Al Darazi, 2020 [22]	France	Phase 1 Trials	Mixed	R;S	259 (153–106)	ICI	Metastatic	Univariate	OS	15 months
Becker, 2020 [23]	USA	Real-World Cohort	Mixed	R;M	122,694 (112,365–10,339)	Mixed	Mixed	Univariate	OS	N/A
Kitadai, 2020 [24]	Japan	Real-World Cohort	NSCLC	R;S	215 (N/A)	ICI	Metastatic	Univariate	OS, PFS	N/A
Nassif, 2022 [25]	France	Phase 1 Trials	Sarcomas	R;M	194 (132–62)	Mixed	Mixed	Multivariate	PFS	35 months
Loh, 2023 [26]	Singapore	Phase 1 Trials	Mixed	R;S	413 (307–106)	Mixed	Metastatic	Univariate	OS	2.3 years
Ma, 2023 [27]	Canada	Real-World Cohort	Gastric-Esophageal	R;S	451 (338–113)	Mixed	Metastatic	Multivariate	OS	9 months

Abbreviations: CRC: colorectal cancer; ICI: immune checkpoint inhibitors; M: multicenter; NSCLC: non-small cell lung cancer; N/A: not available; OS: Overall Survival; P: prospective; PFS: progression-free survival; R: retrospective; RMH: Royal Marsden Hospital; S: single center; SCLC: small cell lung cancer.

**Table 2:** The Newcastle–Ottawa Scale for assessing the quality of studies in meta-analyses.

Author, Year	Selection	Comparability	Exposure/Outcome	NOS Score
Arkenau, 2009 [7]	****	**	***	9
Chau, 2011 [12]	***	**	**	7
Garrido-Laguna, 2011 [13]	***	**	***	8
Hong, 2012 [14]	***	**	***	8
Wherler, 2012 [15]	***	**	***	8
Ferte, 2014 [16]	***	**	***	8
Khan, 2016 [17]	****	**	***	9
Livingston, 2016 [18]	***	**	***	8
Bando, 2017 [19]	***	**	**	7
Sun, 2017 [20]	****	**	**	8
Maymani, 2018 [21]	****	**	**	8
Minami, 2019 [8]	***	*	**	6
Minami, 2020 [9]	***	*	**	6
Al Darazi, 2020 [22]	****	**	***	9
Becker, 2020 [23]	****	**	***	9
Kitadai, 2020 [24]	****	**	**	8
Nassif, 2022 [25]	****	**	**	8
Loh, 2023 [26]	****	**	***	9
Ma, 2023 [27]	****	**	**	8

Each asterisk is equivalent to one point. The maximum score is 9 (**** for selection, ** for comparability, *** for outcome). Score of 5 to 6 considered as moderate quality and 7 to 9 as high quality.

## Data Availability

The data that support the findings of this study are available in the manuscript.
